# Volunteering Transitions and Psychological Well-Being in Family Caregivers of Older Adults

**DOI:** 10.1093/geroni/igaf122.4045

**Published:** 2025-12-31

**Authors:** Sol Baik, Jennifer Crittenden, Kyeongmo Kim

**Affiliations:** University of Virginia, Charlottesville, Virginia, United States; University of Maine Center on Aging, Orono, Maine, United States; Virginia Commonwealth University, Richmond, Virginia, United States

## Abstract

Despite their commitment to caregiving, approximately 25% of family and other unpaid caregivers of older adults in the U.S. participated in formal volunteer activities in 2017. Formal volunteering has been associated with improved psychological well-being, as it offers an additional social role that can enhance self-esteem and expand social networks. However, little is known about how discontinuing volunteering affects caregiver well-being. We hypothesized that ceasing volunteer activity may result in role loss or identity disruption, potentially diminishing psychological well-being, with particular attention to gender differences. Using data from Rounds 5 and 7 of the National Study of Caregiving, we examined the longitudinal effects of starting and stopping formal volunteering on the well-being of 1,148 family and unpaid caregivers of Medicare beneficiaries living in the community. A multilevel mixed-effects generalized linear model was used to assess these associations. Results showed that caregivers who consistently volunteered over time reported significantly better psychological outcomes compared to those who never volunteered. Transitions into or out of volunteering (i.e., starting or stopping) were not significantly associated with changes in well-being overall. However, sensitivity analyses revealed that the positive effects of sustained volunteering were consistent across both women and men. Notably, ceasing formal volunteering was associated with improved psychological well-being among male caregivers. While continued volunteering may generally support caregiver well-being, its psychological impact may vary by gender. For men, discontinuation may offer relief or reprioritization that enhances well-being. These findings highlight the need for gender-responsive strategies to support caregivers through volunteer engagement.

